# A DIGITAL LIFE STORY BOOK ACTIVITY FOR OLDER ADULTS WITH MEMORY DIFFICULTIES: PROGRAM EVALUATION

**DOI:** 10.1093/geroni/igad104.3090

**Published:** 2023-12-21

**Authors:** Elizabeth Barbour, Majse Lind, Susan Bluck

**Affiliations:** University of Florida, Gainesville, Florida, United States; Aalborg University, Aalborg, Nordjylland, Denmark; University of Florida, Gainesville, Florida, United States

## Abstract

Reminiscence activities are based on maintaining a core set of personal memories to aid self-continuity (Woods, 2012). Some interventions find limited effects (Charlesworth et al, 2016) but with participants reporting high satisfaction with the program. The aims of the current study were to: (i) demonstrate feasibility of intervention delivery to older individuals with memory difficulties, and (ii) provide analyses of program efficacy using a pre-posttest, control group design. Participants (N = 45; Mage = 76.12) were randomized to the Life Story Book intervention or Waitlist Control. A Life Story Book Interview was developed, using five evidence-based principles of autobiographical memory. Personalized multimedia books were created and reviewed by participants for fourteen days. To assess feasibility, participants in the intervention completed a memory quality questionnaire and feedback survey. Those who reviewed Life Story Books reported improved memory quality pre-post intervention, t(22) = -2.39, p = .026. Participants also positively rated their intervention experience at post-test, (M = 4.46/5). All participants completed self-related questionnaires (e.g., self-continuity, self-esteem) at pretest-posttest. No measure showed significant effects. Our results represent a conundrum for researchers. We provide evidence of feasibility of, and participant enthusiasm for, this reminiscence-based intervention. As seen in the literature, however, (Elfrink et al., 2021), sensitivity to detect pretest-postest change remains a challenge. The current study highlights participants positive reactions to reminiscence-based Life Story Books. Findings are discussed in terms of methods for assessing intervention efficacy in individuals with memory decline.

